# Feasibility and Safety of 3D-Navigated Trans-Sacral Bar Osteosynthesis for Fragility Fractures of the Sacrum: FIRST Clinical Experiences

**DOI:** 10.3390/jcm13175244

**Published:** 2024-09-04

**Authors:** Stephan Regenbogen, Jan El Barbari, Sven Y. Vetter, Jochen Franke, Paul Alfred Grützner, Benedict Swartman

**Affiliations:** 1 BG Klinik Ludwigshafen, University of Heidelberg, 67071 Ludwigshafen, Germany; stephan.regenbogen@bgu-ludwigshafen.de (S.R.); jan.elbarbari@bgu-ludwigshafen.de (J.E.B.); sven.vetter@bgu-ludwigshafen.de (S.Y.V.); paul.gruetzner@bgu-ludwigshafen.de (P.A.G.); 2 Tauernklinikum, 5700 Zell am See, Austria; jochen.franke@tauernklinikum.at

**Keywords:** intraoperative imaging, sacral bar, surgical navigation, intraoperative computed tomography, image-guided surgery, sacral insufficiency fractures, osteoporotic sacral fractures, fragility fractures of the sacrum

## Abstract

**Background:** There has been an increasing number of fragility fractures of the sacrum in the recent decade. With rates of up to 28%, the complication rates after surgical treatment are still at an unacceptably high level, and new treatment strategies are urgently needed. Therefore, the purpose of this study was to evaluate the potential of 3D-navigated trans-sacral bar osteosynthesis in the surgical treatment of fragility fractures of the sacrum. **Methods:** Retrospectively, from 2017 to 2023, all cases with confirmed fragility fractures of the sacrum in patients > 65 years of age that were surgically treated with navigated 3D-navigated trans-sacral bar osteosynthesis were included, and epidemiological data and the course of treatment analyzed in comparison to a matched control group. **Results:** Finally, 21 patients (18 women and 3 men) were included in this study. The average age of the patients was 82.6 (SD 6.3) in the intervention group and 79.4 (SD 6.7) in the control group. There were postoperatively detected complications in two cases (18%) in the intervention group and in four cases (40%, *p* = 0.362) in the control group. The postoperative in-hospital stay was 10 days (SD 3.8) vs. 11.4 days (SD 3.8) in the control. None of the patients in the intervention group and two in the control group needed revision surgery. **Conclusions**: Overall, 3D-navigated trans-sacral bar osteosynthesis seems to be a promising technique, enabling an accurate implant positioning while offering a low complication rate with an excellent short-term outcome in elderly patients with fragility fractures of the sacrum.

## 1. Introduction

There is an increasing number of low-energy fragility fractures of the sacrum (FFS) which are often accompanied by severe pain, long periods of immobility, and an overall significant decline in physical function leading to an increase in morbidity/mortality [1,2,3,4]. Important risk factors for fragility fracture are osteoporosis and a previous fragility fracture [4]. Therefore, sufficient treatment of the fracture and co-factors like frailty, malnutrition, and osteoporosis is necessary to prevent further fractures.

Overall, treatment algorithms, surgical approaches, type of implant, and postoperative care, have been evaluated and changed repeatedly in the last years. Conservative treatment leads to immobility-related complications, like deep vein thrombosis, pulmonary embolism, and pneumonia in up to 53% of these cases [5,6,7]. Even with operative treatment, there are complication rates involving vascular or neurologic impairment in up to 28% [5,8]. Overall, the optimal treatment regimen for FFS remains controversial and there is no current evidence clearly in favor of one. Thus, treatment is often based on clinical experience, expert opinions, or personal preference.

In general, it is crucial that the osteosynthesis is biomechanically stable under load, and the procedure should be as minimally invasive as possible. In the past, multiple minimally invasive techniques for percutaneous screw positioning of, for example, the sacrum and SI joint, have been developed, resulting in a shorter duration of operation, reduced intraoperative blood loss, and further preserving the surrounding soft tissue compared to open surgery [9]. Therefore, a high amount of posterior pelvic ring injuries can be addressed by minimally invasive SI screw placement. 

Nevertheless, in patients, the sacral anatomy shows an atypical configuration which is called “dysmorphic sacrum”; incorrect placement of SI screws may be associated with neurovascular complications [10]. Rates of screw malposition of up to 25% with a risk for neurovascular complications of up to 18% are described in the literature [11,12,13].

As FFS usually involves both lateral masses of the sacrum and is often associated with an anterior pelvic fracture, bilateral osteosynthesis is necessary. In order to limit soft tissue trauma and the associated morbidity, this is often performed minimal-invasively/percutaneously with iliosacral (IS) screws [14,15]. Due to the reduced bone mass in the elderly, especially in the sacral vertebral bodies of patients with FFS, implant loosening occurs at a rate of up to 20%, and operative revision is needed in up to 18% of these cases [5,14,16]. Therefore, advanced surgical techniques like placement of a trans-sacral bar with washers and nuts placed on both sides or augmented IS screws are used to reduce the risk of implant loosening [3,17,18]. However, the anatomical environment in FFS limits trans-sacral bar positioning with a missing appropriate S1 corridor in almost 50% of the patients [19,20]. The technique of 3D navigation in orthopedic surgery has already been shown to improve both diagnostic and surgical accuracy and, consequently, fracture reduction and implant positioning. Yet, due to the surrounding soft tissues and the complex anatomical situation, correct placement of the trans-sacral bar remains challenging. With intraoperative navigation on the rise, further improvement in surgical accuracy seems to be imminent [21,22,23]. 

Therefore, the aim of this study was to evaluate the feasibility and safety of 3D-navigated trans-sacral bar osteosynthesis for fragility fractures of the sacrum in the elderly. Furthermore, we want to demonstrate our current surgical technique.

## 2. Materials and Methods

This retrospective study was performed at a single, level-one trauma center. The ethical committee of the Medical Association of Rhineland-Palatinate (Germany) under research no. 2024-17414 approved this study. Consecutively, all cases with fragility fractures of the sacrum (FFS) injury which were treated by 3D-navigated trans-sacral bar osteosynthesis of the sacrum between January 2017 and December 2023 were evaluated. The eligibility criteria for enrollment in this retrospective analysis were an age above 65 years, and patients with minor trauma. Excluding criteria for this study were major trauma, i.e., polytrauma or high energy impact. According to the inclusion and exclusion criteria, a matched control group was established using 10 cases in which 3D-navigated sacroiliac joint osteosynthesis was performed at our institution.

The following variables of the final cohort were extracted and analyzed: patient’s age, sex, disease profile, trauma mechanism, time to surgical intervention, fracture classification, and accompanying fractures, operation time and blood loss, complications and revision, and in-hospital stay. Data are shown and summarized in Table 1.

Furthermore, mobility and social independence before the injury and after the hospital treatment course were evaluated. The fracture type was analyzed using computed tomography (CT) and classified using the Fragility Fractures of the Pelvis classification (FFP) by Rommens et al. [24]. 

### 2.1. Surgical Technique and Postoperative Treatment

**Figure 1:**
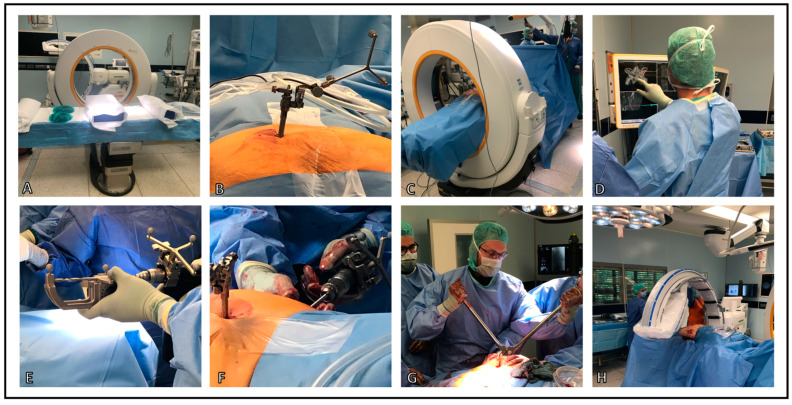
Intraoperative Setting and Workflow. (**A**) Setup of the operating table, intraoperative CT (iCT), and C-arm prior to the procedure. (**B**) Initially, the reference base for the CT scan and navigation system is placed on the dorsal iliac crest. (**C**) Acquisition of the iCT scan. The drapes are taped below the table to prevent entanglement. (**D**) Planning of the implant size and trajectory by the surgeon. (**E**) Calibration device for the tools and implants before usage. (**F**) Percutaneous placement of the trans-sacral bar. (**G**) After the counterlock has been fastened, the overlapping part of the bar is shortened. (**H**) Final fluoroscopic control and documentation of implant placement and reduction.

Intraoperative Setting begins with placement of the operating table, intraoperative CT, and C-arm prior to the procedure (Figure 1A). Surgery is performed under general anesthesia and started in the prone position. After a team time-out, a reference base for 3D navigation is attached to one side of the posterior iliac crest after a stab incision (Figure 1B). The base is used to perform either a referenced 3D scan (Cios Spin, Siemens Healthineers, Forchheim, Germany) of the posterior pelvic ring or, if there is insufficient volume, a referenced CT scan (Airo, Brainlab, Munich, Germany) of the pelvis. Laser guidance is employed to determine the extent of the surgical field. Acquisition of the scan is performed. The drapes are taped below the table to prevent entanglement (Figure 1C).

The navigated pointer is used to aim at the desired trajectory (Curve, Brainlab, Munich, Germany) in S1 or S2, and the skin incision is made accordingly. After blunt dissection of the bone, the bone surface is verified with the Pointer tip. The desired bi-transiliac-transsacral trajectory and implant size are now planned virtually (Figure 1D). Next, a calibration device for the tools and implants is used (Figure 1E).

**Figure 2:**
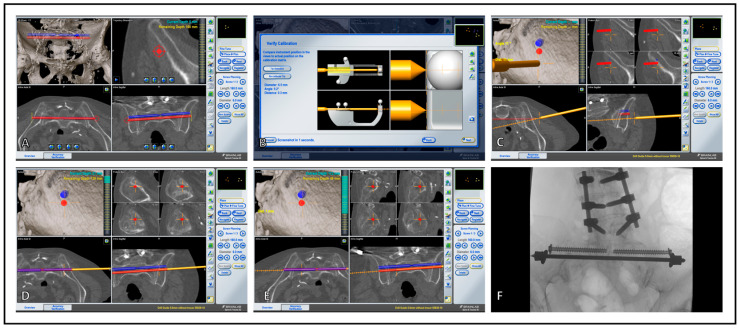
Software workflow of the navigated placement of a trans-sacral bar. (**A**) After acquiring the intraoperative navigation scan, planning the trajectory is possible with the assistance of a pelvic 3D rendering along with sagittal, coronal, and axial plane displays. The length and diameter of the sacral bar, as well as the placement of additional SI screws, can be adjusted. (**B**) Following the planning of trajectories and implants, the calibration of the tools and implants is verified. (**C**) During implant insertion, any deviations from the insertion point in the bullet view, as well as deviations from the trajectory axis, are displayed. Additionally, the currently aimed trajectory is shown in the three CT planes. (**D**) Once the correct entry point and trajectory are achieved, the sacral bar can be inserted, with visual feedback on the progression of the insertion. (**E**) After placing the sacral bar, additional SI or acetabular screws can be inserted as needed. (**F**) Finally, intraoperative fluoroscopy is used to assess and document the placement of implants.

For percutaneous placement of the trans-sacral bar (Figure 1F), a navigated drill sleeve is calibrated. A guide wire is placed according to the planned trajectory. If necessary, the position can be checked with radiographic imaging in two planes or with an additional 3D scan. If the wire position is satisfactory, it is drilled over with a 6 mm cannulated drill. A solid 6 mm threaded rod (Johnson & Johnson, New Brunswick, NJ, USA) is advanced in the pre-drilled canal after the removal of the wire and under fluoroscopic control. A contralateral stab incision is made above the end of the rod. The rod is secured on both sides with a washer, nut, and lock nut. Exceeding parts of the rod are cut off with a bolt cutter (Figure 1G). Depending on the fracture type, additional navigated large fragment cannulated screws (Johnson & Johnson, New Brunswick, NJ, USA) are placed as anti-rotation screws in S1 or S2. Finally, the result is documented by X-rays in two planes (Figure 1H). Postoperative, all patients are allowed full weight bearing as tolerated under physiotherapy control. The surgical procedure for 3D-navigated sacroiliac joint osteosynthesis is performed accordingly; a detailed procedural description can be found in a preliminary study [25]. A step-by-step illustration of intraoperative Setting and Workflow is presented in Figure 1 and Figure 2.

### 2.2. Statistical Analyses 

Descriptive statistics, including means, frequency counts, percentages, and ranges were determined as appropriate for continuous and categorical variables. Descriptive statistics were expressed as mean values with standard deviation and percentages. 

Groups were compared using Fisher’s exact test for categorical variables or Chi-squared test in cases of more than two outcomes, and a Welch ANOVA with Dunnett T3 correction for multiple testing for the interval-scaled variables.

Statistical analyses were performed, and graphics were designed using the Prism program from GraphPad Software (San Diego, CA, USA), version 8.3.1. Statistical significance was set at *p* < 0.05. Due to the small sample sizes, statistical analyses are exploratory in nature and not confirmatory.

## 3. Results

**Figure 3:**
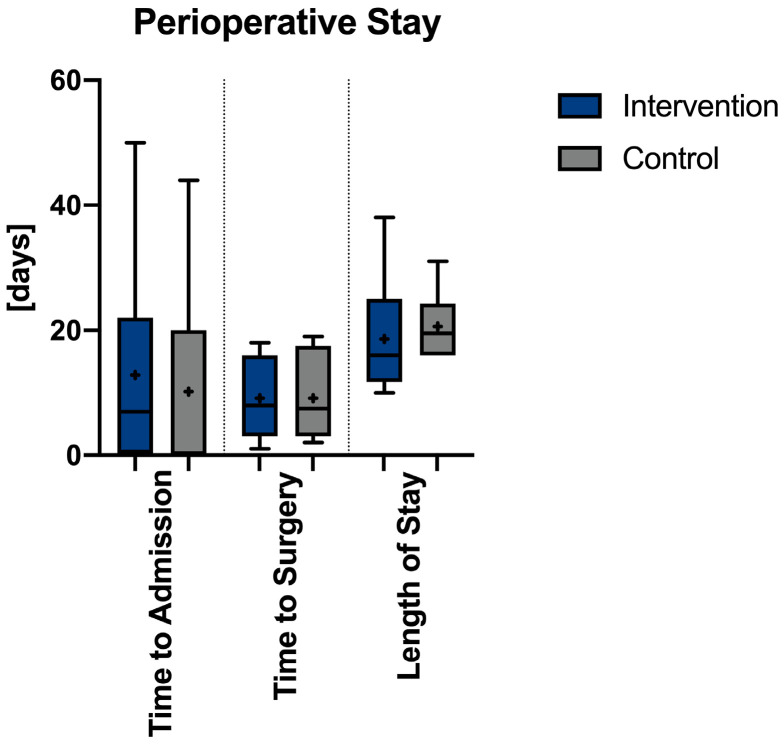
Comparison in perioperative stay between the intervention and control group. The box-plots are designed with whiskers reaching from minimum to maximim with the + indicating the mean and the horizontal line the median.

Finally, 21 patients (18 women and 3 men) were included in this study. The average age of the patients was 82.6 (SD 6.3) in the intervention group and 79.4 (SD ± 6.7) in the control group (95% CI −6.19; 12.59). In the intervention group, in 82% of the cases (9/11), it was a bilateral injury, in the remaining 18% (2/11), only the left side was involved. In the control group, in 60% (6/10) a bilateral fracture occurred while, in three cases, it was only affecting the left, and in one case the right side. The difference was not statistically significant (*p* = 0.552). Fractures in the intervention group were mostly caused by minor trauma, like falling from a standing position (*n* = 8, 73%); in three patients (27%) no relevant primary trauma could be found. In the control group trauma mechanism was minor in eight cases (80%); in two patients (20%) no relevant primary trauma could be found without reaching statistical significance (*p* = 0.214). The mean time from trauma/symptoms to first presentation was 12.9 days (SD17.2) in the intervention group, while, in one case, the time could not be determined, while the mean delay until admission was 10.2 days (SD 16.2) in the control group (*p* = 0.999, 95% CI −21.35; 26.75, see Figure 3). The mean BMI was comparable, with a mean of 26.2 (SD 4.1) in the intervention group and 27.5 (SD 6.6) in the control (*p* = 0.997, 95% CI −9.58; 6.984) (see Table 1).

### 3.1. Fracture Classification

**Figure 4:**
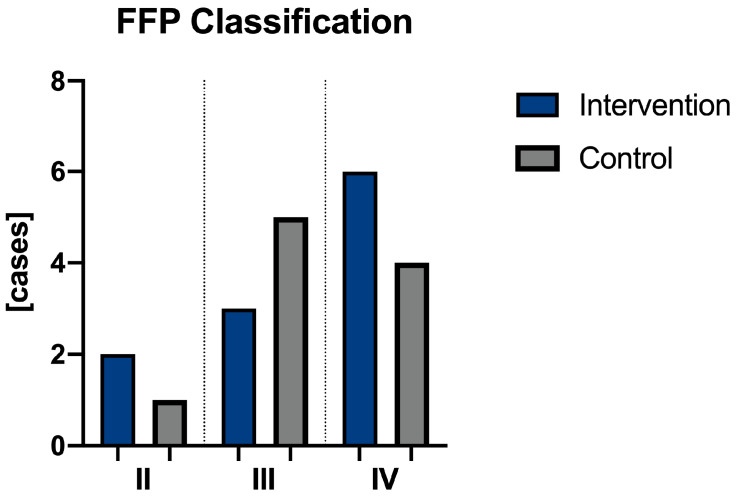
Comparison of FFP classification according to Rommens et al. [24] between the intervention and control group.

According to the classification of Rommens et al. [24], in the intervention group, Type IV was found to be the most common one in six patients (55%), followed by Type III in three patients (27%) and Type II in two cases (18%). Concomitant injuries to the pelvic ring were found in five cases (45%). In the control group, Type III was found to be the most common one in five patients (50%), followed by Type IV in four patients (40%), and Type II in one case (10%). Concomitant injuries to the pelvic ring were found in six cases (60%). We could not find any statistical significance regarding the type of fracture between the groups (Figure 4). 

### 3.2. Comorbidities

Confirmed Osteoporosis was found in seven cases of the intervention group (64%) and six patients in the control group (60%). All patients in the intervention group and nine patients in the control group had at least two concomitant diseases. Cardiologic pathologies were the main concomitant diseases with nine cases (82%) in the intervention group and eight cases (80%) in the control group. These differences were not statistically significant (see Table 1).

### 3.3. Duration of Surgery and Blood Loss 

Operative treatment was performed, comparably, at a mean of 9.2 days (SD 6.3) after hospital admission in the intervention group and 9.2 days (SD 6.7) in the control group (*p* > 0.999). With an average duration of surgery of 166 min (SD 73) in the intervention group and 142 min (SD 68) in the control, no significant difference could be found here as well (*p* = 0.977, 95% CI −77.53; 125.5). Intraoperative blood loss was <500 mL in all cases of the intervention group and nine (90%) of cases in the control group (*p* > 0.476).

### 3.4. Hospital Stay 

The average length of hospital stay was 18.6 days (SD 8.8) in the intervention group compared to 20.6 days (SD 5.0) in the control, while the average length of postoperative hospital stay was 10 days (SD 3.8) vs. 11.4 days (SD 3.8) in the control. Both differences were not statistically significant (p_total_ = 0.992, p_postoperative_ = 0.966, 95% CI −6.88; 4.08).

### 3.5. Complications and Revisions 

**Figure 5:**
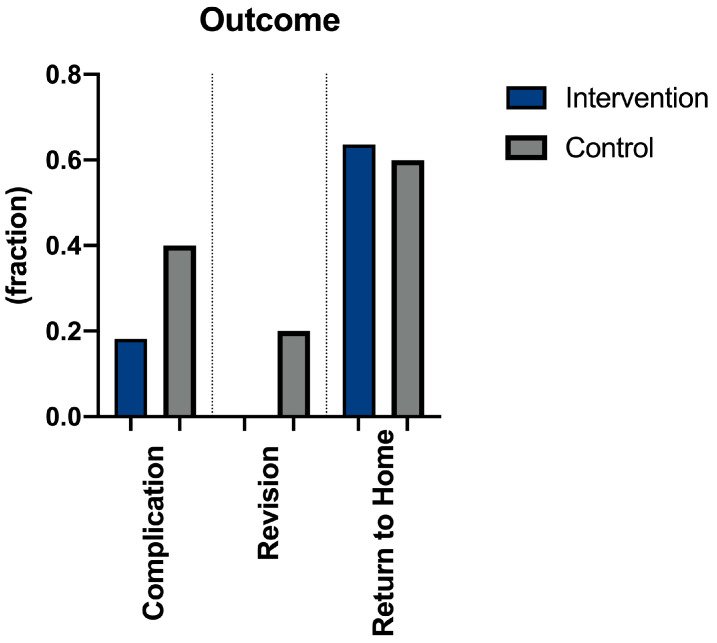
Comparison in complication, revision, and return to the previous stage between the intervention and control group.

In the intervention group, the number of postoperatively detected complications after using 3D-navigated trans-sacral bar osteosynthesis was two cases (18, 8%), including one with bleeding and death. The one death reported was due to heart failure. No operative revision was necessary. No implant failure or loosening was observed. In the control group, complications occurred in four cases (40%, *p* = 0.362). In one patient, a secondary increase of dislocation of the accompanying pelvis fracture due to minor trauma was found. One had cardiopulmonary instability due to postoperative bleeding requiring circulatory medications and blood transfusions. The other complications included one case of postoperative cement leakage intraspinally and one case of prerenal kidney failure. Two cases (20%) needed operative revision due to implant failure and revision of hematoma (95% CI −12.44; 8.44, see Figure 5).

### 3.6. Discharge from Hospital Stay 

Most patients were able to return to their previous stage of the social environment (*n* = 7, 70%), while three patients (30%) needed new or increased additional support in the intervention group. This was the case in only six cases (60%) in the control group (see Figure 5).

## 4. Discussion

Overall, there is a significant increase in age at incidence in elderly patients. Due to increasing life expectancy, with a doubling of people over 80 years of age in 2040, a further increase in FFS is also to be expected [26].

FFS is often accompanied by severe pain, long periods of immobility, and overall a significant decline in physical function [1,2,3,4] which leads to a mortality rate of up to 30% [5,27]. Choosing the appropriate treatment regime is very important as the most important risk factor for fragility fracture is a previous fragility fracture [4]. Conservative treatment leads to immobility-related complications in up to 53% [5,6,7], but also, with surgical treatment, high complication rates of up to 28% occur [5]. Overall, the treatment regimens for FFS are still controversial and there is no current evidence.

FFS often involves both sides of the sacrum and therefore a bilateral osteosynthesis is necessary, which is often performed in a minimal-invasive/percutaneous manner, with iliosacral (IS) screws [14,15]. However, implant loosing occurs in up to 20% of cases, and operative revisions are needed in up to 18% [5,14,16]. Therefore, advanced surgical techniques, like a trans-sacral bar with both-sided washer and nuts or augmented IS screws, are used to reduce the rate of implant loosening [3,17,18]. Furthermore, trans-sacral screw stabilization could reduce the risk of implant loosening because it provides more stability than bidirectional IS screws [3,28]. Furthermore, operative treatment with trans-sacral bar was significantly associated with a shorter preoperative and total length of hospital stay and a lower mortality rate [5]. Nevertheless, the anatomical environment in FFS limits trans-sacral bar positioning in almost half of the patients [19,20]. 

The technique of 3D navigation in orthopedic surgery has been shown to provide better intraoperative imaging quality and control of reduction and implant position compared to 2D navigation or fluoroscopic control and may increase the accuracy and safety of operative procedures [21,22,23]. 

Our study aimed to evaluate the feasibility and safety of 3D-navigated trans-sacral bar osteosynthesis for FFS in elderly patients, comparing it to a control group treated with 3D-navigated bilateral sacroiliac joint osteosynthesis. 

In the case of a dysmorphic sacrum, particularly while relying solely on fluoroscopic imaging [19,20], it can be impossible to fit a transverse bar into the S1 corridor due to its ascending character. If this is so, the implant has to be placed in the S2 corridor. It must be noted that this corridor is significantly narrower and the bone in the sacral body weaker. The technique of 3D navigation seems to be a helpful tool in order to place the bar in the narrow S2 corridor in these cases. Marintschev et al. introduced a bilateral fixed-angle sacral nail and reported no implant-associated complications and no inpatient complications in 27 cases [29]. In one case, additional plating of the anterior pelvic ring was necessary later. Our study supports these findings, as no cases of implant loosening were observed, even among the 64% of patients with confirmed osteoporosis.

Since bleeding, though rare, was a relevant complication in our study, it must be assumed that opening the soft tissues in order to cut the bar at the right length could be a critical maneuver. The positioning of the patient also has to be in the prone position. This could be avoided by, for example, using the Axomed sacral bar, which can be applied in the supine position. This positioning allows additional one-stage plating of the anterior pelvic ring, if necessary.

The duration of surgery in our study took 166 min for the sacral bar, whereas 142 min were necessary in the control group, whilst Kramer et al. reported 53 min for two monoportal transsacral screws [14] and Marintschev 130 min for the sacral nail [29]. Though surgery seems to be prolonged by using 3D navigation and the necessity of a bilateral approach, however small in this percutaneous technique, the disadvantage of the monoportal transsacral screws is, in our opinion, the loosening rate of 19% [14] compared to 0% in our intervention group and one (10%) in the control group. 

There are techniques described without navigation as by Wagner, although these result in revision rates of 15% [5]. It can be discussed whether the navigated technique prevents implant-related complications due to higher precision and perhaps smaller incisions. While the intraoperative CT offers a sufficient volume to display the entire posterior pelvic ring (Kramer), it involves extensive preparation. We experienced that a cone beam CT, as is obtained by the flat panel C-arm (Cios Spin, Siemens Healthineers, Forchheim, Germany), creates an adequate volume to safely navigate the sacral bar in the posterior pelvic ring as well.

Most importantly, though the intervention group showed a tendency towards a higher complication and revision rate and the necessity for a new or increased additional support compared to the intervention group. 

Thus, the placement of a 3D-navigated trans-sacral bar osteosynthesis for fragility fractures of the sacrum appears to be a feasible option in elderly patients, promoting a faster return to mobility, reducing the length of hospital stay, and minimizing post-operative complications.

## 5. Conclusions

Taken together, we demonstrated that our surgical technique with 3D-navigated trans-sacral bar osteosynthesis for fragility fractures of the sacrum in the elderly is a feasibility and safe operative treatment option allowing an accurate control of reduction and implant positioning, and therefore may increase safety in operative treatment of FFS. It can be assumed that the time of operation will be reduced with increasing experience and expertise in workflow and operative procedure. Furthermore, from our current perspective, besides enabling an accurate implant positioning, the biomechanics of a continuous implant could presumably be superior to those of bilateral screw fixation and, thus, there could be significantly fewer rates of implant loosening, even in patients with osteoporosis. Nevertheless, further studies with larger cohorts and randomized prospective studies with bigger matching comparison groups are urgently needed to critically evaluate the safety of 3D-navigated trans-sacral bar osteosynthesis for fragility fractures of the sacrum in comparison to the existing surgical treatment options. 

## 6. Limitations

- This study has all the inherent weaknesses that accompany a retrospective study.
- Only the assessed and documented data of the patients were available since no clinical follow-up (validated outcome scores or PROMs) was performed.
- The main limitation of this study is the small sample size. Therefore, this conclusion can be only seen as a mere implication and further data are necessary to support this claim.
- Delayed complications were probably underestimated due to the short follow-up, but they were not the objective of our study.
- Fracture healing and patient mobility should be assessed in further investigations.

## Figures and Tables

**Table 1:** Patient demographics and outcome variables values are presented in n (%) if not mentioned otherwise. ^F^ Fisher’s exact test, ^C^ Chi-squared test, ^t^ Welch-ANOVA with Dunnett T3 correction, Statistical significance with *p* < 0.05.

Cases	Intervention 11 (52.4)	Control 10 (47.6)	*p*-Value
Age (years), mean ± SD	82.6 ± 6.3	79.4 ± 6.7	0.0869 ^t^
Sex (male)	1 (9)	2 (20)	0.587 ^F^
BMI, mean ± SD	26.2 ± 4.1	27.5 ± 6.6	0.997 ^t^
Osteoporosis (yes)	7 (64)	6 (60)	>0.999 ^F^
Comorbidities (>2)	11 (100)	9 (90)	0.476 ^F^
Minor trauma (yes)	8 (73)	8 (80)	0.214 ^F^
No trauma (yes)	3 (27)	2 (20)	>0.999 ^F^
Bilateral injury (yes)	9 (82)	6 (60)	0.362 ^F^
Fracture Classification (FFP)			0.552 ^C^
II	2 (18)	1 (10)	
III	3 (27)	5 (50)	
IV	6 (55)	4 (40)	
Time to Admission (days), mean ± SD	12.9 ± 17.2	10.2 ± 16.2	0.999 ^t^
Time to Surgery (days), mean ± SD	9.2 ± 6.3	9.2 ± 6.7	>0.999 ^t^
Length of total stay (days), mean ± SD	18.6 ± 8.8	20.6 ± 5.0	0.992 ^t^
Postoperative stay (days), mean ± SD	10.0 ± 3.8	11.4 ± 3.8	0.966 ^t^
Duration of surgery (min), mean ± SD	166 ± 73	142 ± 68	0.977 ^t^
Intraoperative blood loss (<500 mL)	11 (100)	9 (90)	0.476 ^F^
Complication	2 (18.8)	4 (40)	0.362 ^F^
Revision	0 (0)	2 (20)	0.214 ^F^
Return to previous home (yes)	7 (70)	6 (60)	>0.999 ^F^

## Data Availability

Data are unavailable due to privacy or ethical restrictions.
